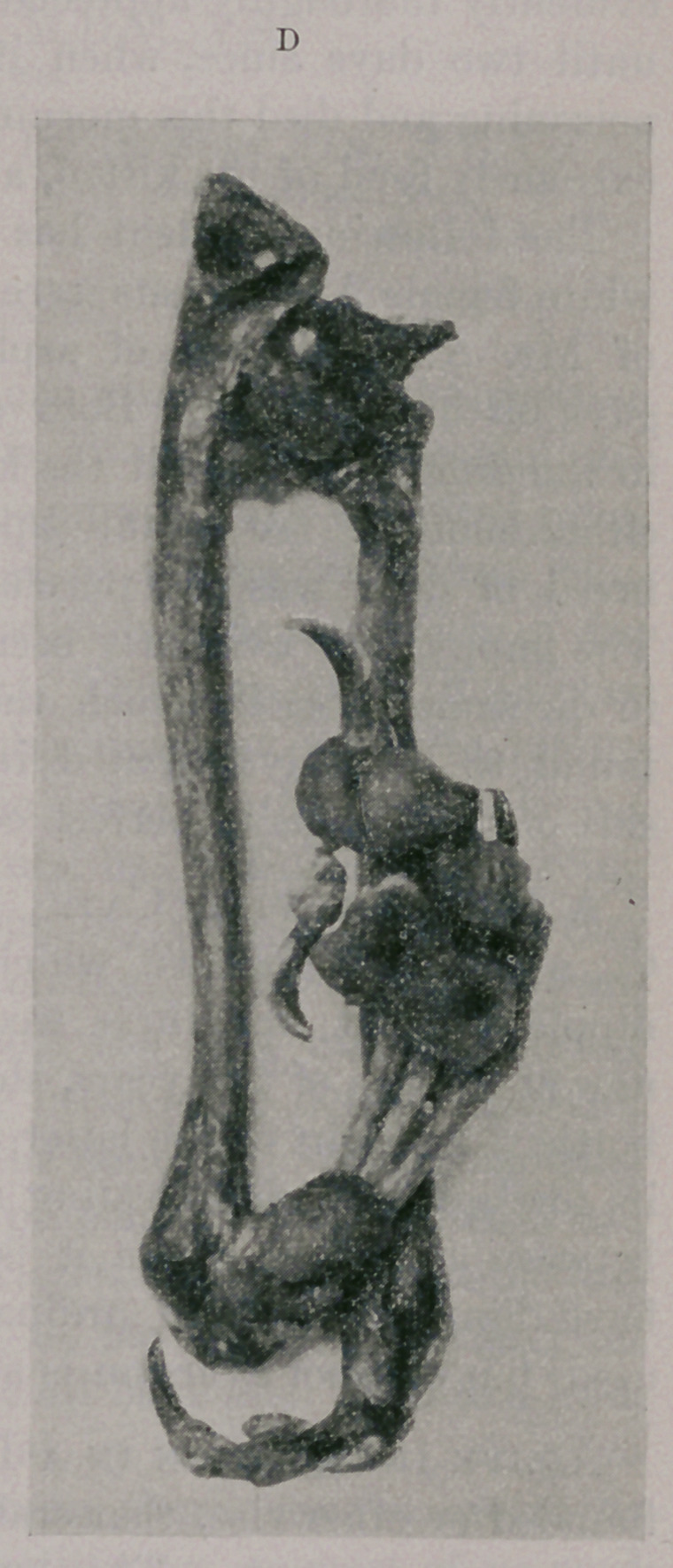# Department of Canine and Feline Medicine and Surgery

**Published:** 1900-09

**Authors:** Cecil French

**Affiliations:** Washington, D. C.


### DEPARTMENT OF CANINE AND FELINE MEDICINE AND SURGERY.

BY CECIL FRENCH, D.V.S.,

WASHINGTON, D. C.

### CARPUS UNILATERALIS CONGENITALIS ET LUXATIO CAPITI RADII (CONGENITALIS?).

UNDER this term I shall describe a congenital deformity in a dog which was recently brought to my notice by Lincoln Johnston, M.D., of this city. Dr. Johnston obtained the specimen at a local pound, having severed the left leg at the inferior extremity of the humerus, after the animal, which was a full-grown mongrel and weighed some fifty pounds, had been removed from the lethal chamber. Unfortunately, he did not see the animal in the living state, so did not observe its mode of progression.

The condition and relative position of the parts in this specimen correspond to what is known in the human subject as "talipes," or club-foot.

Gould defines talipes as: "Club-foot, a deformity depending upon contraction of one or more muscles or tendons about the foot, either congenital or acquired," the word being derived from *talus*, an ankle, and *pes*, a foot. The deformity in my case having existed in the foreleg, it became necessary, in order to be specific in my definition, to draw on the word *carpus* instead of *talus*. In the human subject the congenital form has been attributed to deficiency in the amount of liquor amnii. This would cause compression of the feet by the uterine walls, leading to contraction of the ligaments and muscles on one side and stretching of those on the other, the condition becoming permanent. Another cause is given as disordered nerve-function acting on certain muscles which would draw the foot into the deformed position.

Photographs A and B represent the specimens as received by me. It is shown here in the vertical position with the sides exposed to view, which was evidently the manner in which the leg was made to support the body when at rest during the animal's life. But the appearance of the pads of the ruptured digits, indicating that they had been subjected to ordinary wear, inclined me to the opinion that the movements of this leg had been conducted in an antero-external oblique direction.

The specimen was dissected under the supervision of Dr. Lamb, pathologist at the Army Medical School, and deposited in the museum of that institution, after again being photographed, as represented in pictures C and D.

A

B

The ulna is in normal position and articulates properly with the distal extremity of the humerus. The radius is completely separated from the ulna, its head being in a position of upward dislocation and antero-internal rotation, with resultant pseudoarthrodic formation. Whether this humero-radio-ulnar dislocation was of congenital origin or not it is difficult to say. The weight of the body seemed to have been largely borne by the radial portion of the irregularity, which may have been sufficient to have produced a luxation after birth.

Both portions of the carpal joint were almost immobile. The component bones were not isolated, since this would have necessitated such distinction as to destroy the specimen for exhibition purposes ; but it can be seen that those articulating with the ulna give rise to three perfect digits, and those forming the radial por-

C D

tion one perfect digit. Attached to one of the three digits was an imperfect one formed of its distal phalanx only.

Photographs A and B give a good idea of the displacement these bones have undergone.

THE CONDITIONS REVERSED. In the *Veterinarian*, 1896, vol. lxix., p. 683, is the following extract from *The Field* (English), of August 15th, headed : " A Precocious Puppy." " About four months since we had a mongrel bitch puppy given us, about the size of an ordinary fox-terrier, and she is seven months old at the present time. Two months since we had a male kitten given us about five weeks old, which its mother had deserted. About a week after having had it we noticed that the puppy was full of milk, and could not understand it, until one day we caught the kitten lying down and sucking the bitch puppy, which the latter evidently thoroughly appreciated. The kitten continued to do so until two days since, when it showed signs of sickness through poisoning and died this morning. I may add that the puppy was extremely fond of the kitten, and it showed signs of intense grief."

The following incident has recently come to my notice: Two white female Angora cats were acquired by a member of the family of Mr. A. L. Barber, of asphalt fame, and not long after both gave birth to kittens. Both of the cats and their families nested in the same box, and all the kittens nursed indiscriminately from either mother. A minute specimen of the Spanish silk poodle breed of dogs was also purchased after the birth of the kittens. The puppy had evidently been only just weaned. It was housed in the same room in which the cats were, and before long it was found to be fully affiliated into the feline circle, suckling alongside of the kittens in perfect contentment.

A Bottle-loving Cat. Miss Purman, of Takoma Park, D. C., owns an Angora cat which she raised artificially by bottle and nipple since it was three weeks old. This particular animal, now at the age of two years, shows a remarkable love for the milk-bottle. At sight of the latter it rolls over on its back and extends its forelegs in a supplicating manner. The bottle is then placed between its paws, where it is safely supported until the contents have disappeared. An ordinary baby's feed-bottle and nipple are used, but distinct aversion is shown to a new nipple.

Acute Indigestion in a Dog. There is a popular idea, well founded or otherwise, that crab-meat and milk are incompatible in the human stomach. They would certainly seem to be in that of the dog. A year-old fox-terrier made an ordinary meal of these two article of diet, and within ten minutes was seized with clonic spasms. The spasms continued some thirty minutes, until he was brought to me. Following my usual course of procedure in spasm cases, and without having been informed as to the foodstuffs recently taken, I administered a hypodermatic injection of muriate of apomorphine, grain one-twentieth, and followed this up with rectal injections of water to stimulate evacuation of the bowels. Emesis was promptly produced, the vomitus consisting of curdled milk, unchanged crab-meat, mucus, and digestive fluid. The nervous symptoms then rapidly subsided, and within half an hour the animal was returned to its owner.

## Figures and Tables

**A f1:**
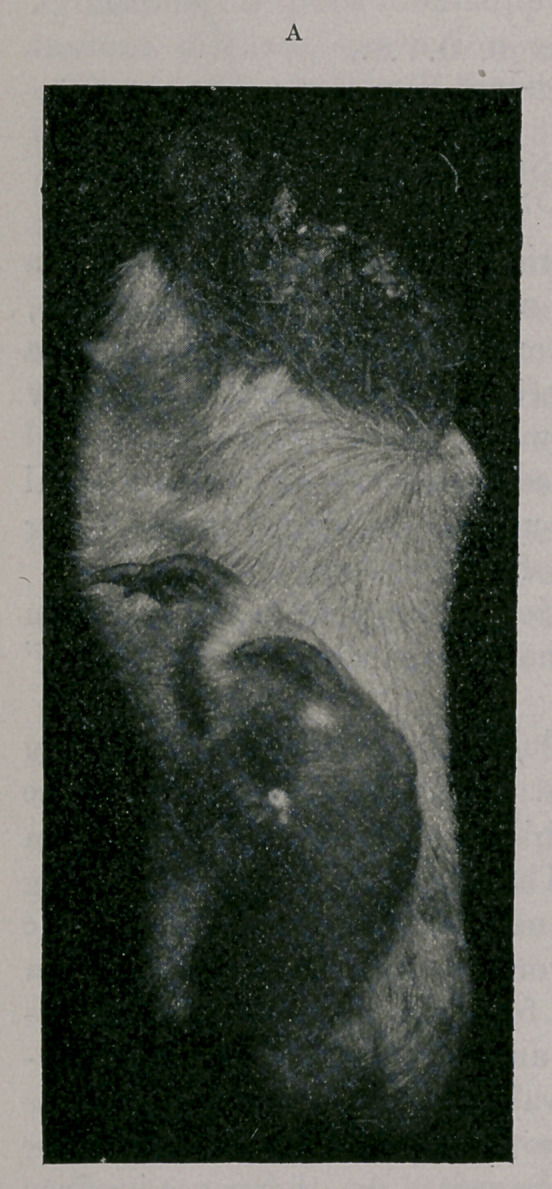


**B f2:**
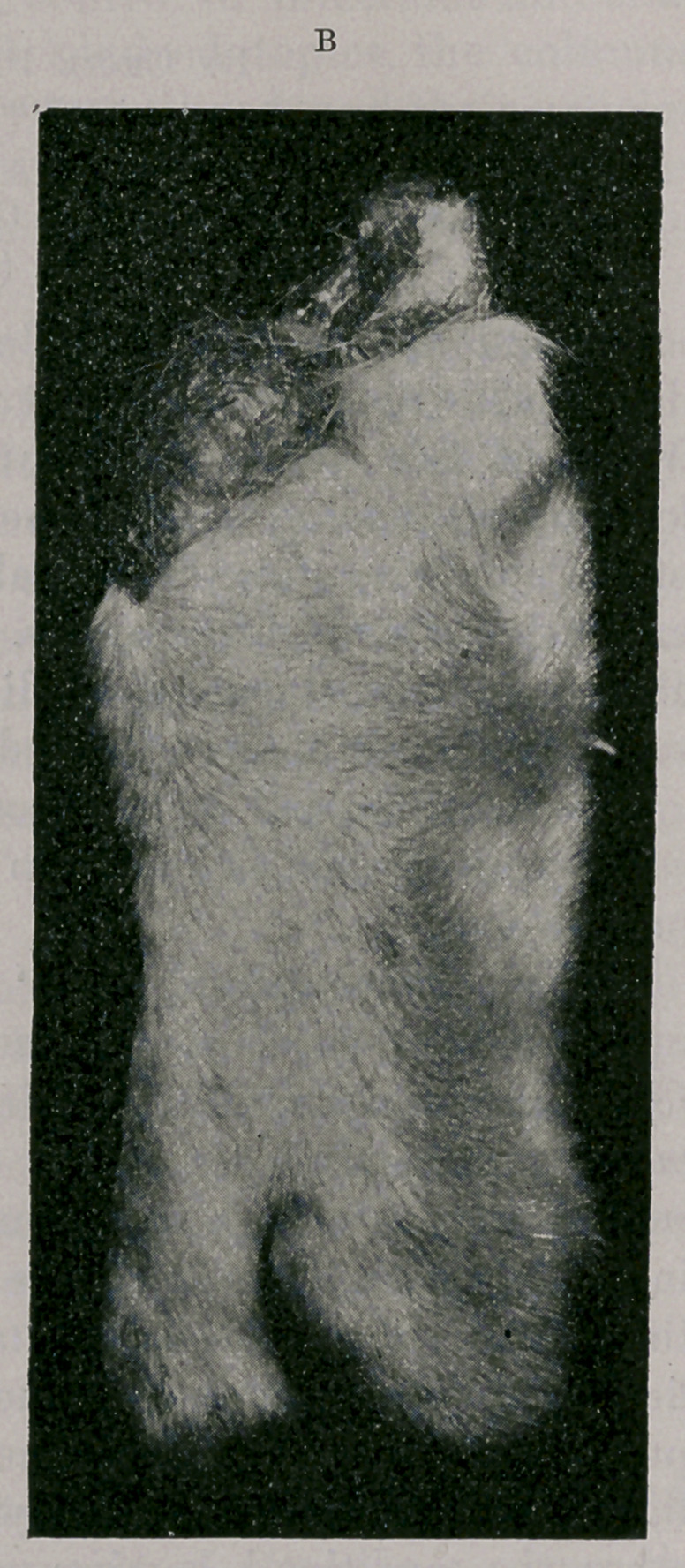


**C f3:**
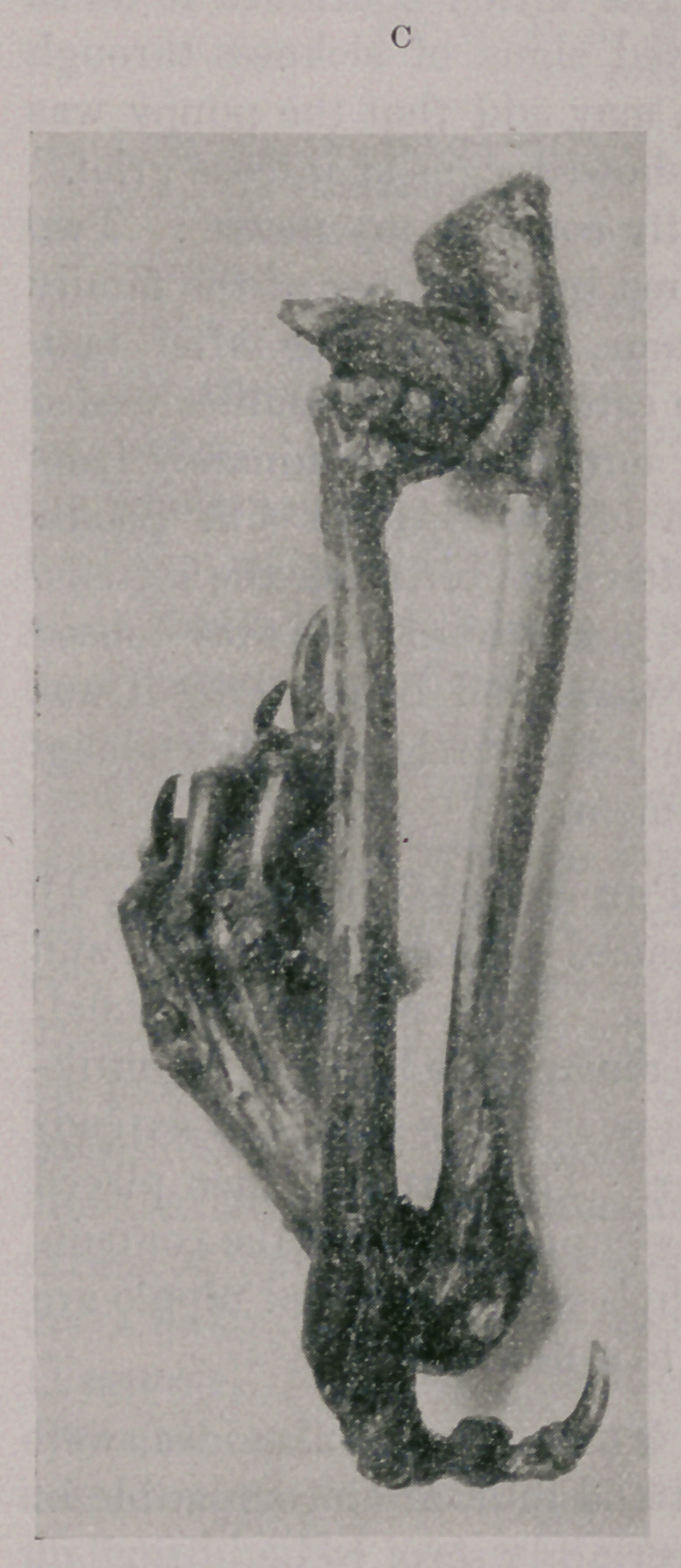


**D f4:**