# Correction

**Published:** 1900-10

**Authors:** 


CORRECTION. In Dr. French's paper on page 543, September number, by error of the printer, "Carpipes Unilateralis," etc., was made to read "*Carpus* Unilateralis," etc.

